# Discovery of the High-Affinity Aptamer for Candidalysin Using a Dual-Mode Colorimetric–SERS Platform

**DOI:** 10.3390/bios16010035

**Published:** 2026-01-02

**Authors:** Yige Sun, Canlan Zheng, Yuxuan Shi, Mingyuan Sun, Chao Wang, Lin Han, Yu Zhang, Tiezhou Hou, Le Qiang

**Affiliations:** 1 Key Laboratory of Shaanxi Province for Craniofacial Precision Medicine Research, College of Stomatology, Xi’an Jiaotong University, Xi’an 710004, China; sunyige@stu.xjtu.edu.cn (Y.S.); 418524572@stu.xjtu.edu.cn (C.Z.); 2206124511xuan@stu.xjtu.edu.cn (Y.S.); 2 Clinical Research Center of Shaanxi Province for Dental and Maxillofacial Diseases, College of Stomatology, Xi’an Jiaotong University, Xi’an 710004, China; 3 Department of Cariology and Endodontics, College of Stomatology, Xi’an Jiaotong University, Xi’an 710004, China; 4 Institute of Marine Science and Technology, Shandong University, Qingdao 250100, China; sunmingyuan@sdu.edu.cn (M.S.); wangchaosdu@sdu.edu.cn (C.W.); hanlin@sdu.edu.cn (L.H.); yuzhang@sdu.edu.cn (Y.Z.); 5 Department of Integrated Circuits, Shandong University, Jinan 250100, China

**Keywords:** Candidalysin, high-affinity aptamer, dual-mode, biosensor, AuNPs

## Abstract

*Candida albicans* poses significant health risks through its virulent peptide toxin Candidalysin. As no existing therapeutics specifically target this toxin, developing high-affinity aptamers for its efficient and safe removal is urgently needed. In response, we developed a dual-mode biosensor based on gold nanoparticles (AuNPs) and aptamers for screening high-affinity aptamers for Candidalysin. This biosensor leverages the localized surface plasmon resonance (LSPR) phenomenon and surface-enhanced Raman scattering (SERS) of AuNPs to detect changes in color and Raman signals, respectively, indicative of high-affinity aptamer for Candidalysin presence. This dual-mode capability reduces false-negative signals and enhances detection accuracy. Our findings reveal a specific aptamer with high affinity for Candidalysin, presenting a significant advancement in candidiasis treatment. This work sets the stage for the development of effective therapeutic strategies against Candida infections.

## 1. Introduction

Fungal infections are notoriously refractory and formidable, causing severe complications and significant mortality rates [1,2]. *Candida albicans*, a common opportunistic pathogen colonizing the human oral cavity, skin, and mucous membranes, causes both local and systemic infections [3,4,5]. The transition of *C. albicans* from yeast to invasive hyphae leads to severe epithelial damage [6,7]. In 2016, Moyes et al. reported the discovery of Candidalysin, a virulent peptide toxin secreted by hyphae, triggering adverse host cell activation during epithelial infection, which has the ability to damage epithelial membranes, activate adverse response signal pathways, and engage epithelial immunity establishment [8]. This peptide toxin, identified as the first in human fungal pathogens, plays a crucial role in candidiasis infection [9]. The efficient, harmless, rapid, and precise removal of Candidalysin is key to candidiasis treatment. However, there are no small-molecule compounds with high affinity for Candidalysin that have yet been found.

The unique optical properties of nanomaterials enable sensitive detection of proteins, peptides, ctDNA, miRNA, exosomes, and CTCs [10,11]. The label-free colorimetric biosensor, based on aptamers and gold nanoparticles (AuNPs), is currently a high-performance biosensor for detecting small molecules [12,13]. It operates on the localized surface plasmon resonance (LSPR) phenomenon of AuNPs [14,15]. AuNPs change from a monodisperse state to an aggregated state, inducing a visible color change from red to blue. Upon adding a certain amount of aptamer to the aggregated AuNPs, aptamers adsorb on the surface of AuNPs through physical forces such as van der Waals forces and hydrogen bonds [16], then the colloid restores charge balance and the color reverts to red. The introduction of a target with high-affinity for the aptamer causes separation of aptamers from the surface of the AuNPs, then the AuNPs aggregate again and the color shifts from red to blue [17,18,19]. Besides the color change, AuNPs can also be used for surface-enhanced Raman scattering (SERS) sensing by modulating the interparticle distance between Raman molecule-modified AuNPs, owing to the simultaneous contribution of plasmon resonance and a Raman molecule [20,21]. The detection sensitivity of SERS can be dramatically enhanced by 10^2^ to 10^14^ times compared with conventional Raman scattering [22]. Lately, there are many dual-mode biosensors based on these two principles for rapid detection of pathogens, which can greatly reduce false-negative signals and improve detection accuracy. For example, Sha Liu et al. constructed a facile dual-mode aptasensor for tetrodotoxin [23], Zhengzong Wu et al. used a colorimetric–SERS dual-mode method to specifically detect *Pseudomonas aeruginosa* [24], Kexin Ren achieved the detection of Shiga toxin type II by a colorimetric and SERS dual-mode aptasensor [25], and Nan Zhang et al. developed a colorimetric–SERS dual-mode biosensor capable of detecting SDM within 20 min [26]. A dual-mode biosensor is a viable approach for high-affinity aptamer screening for Candidalysin.

**Figure 1:**
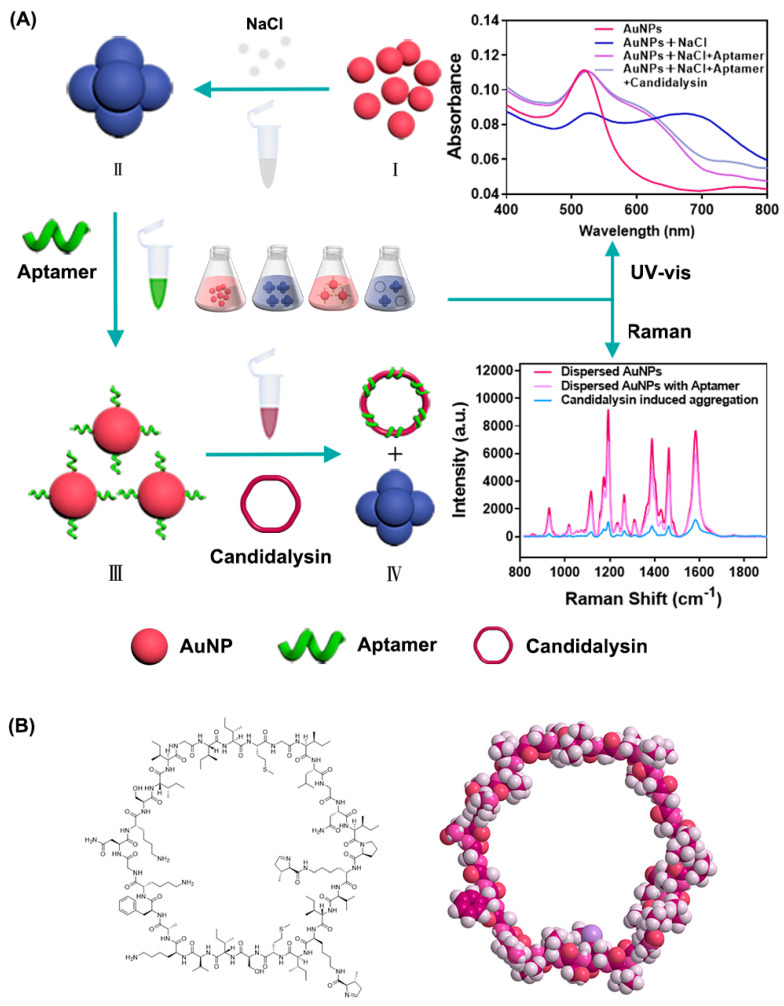
(**A**) Schematic diagram of the colorimetric biosensor. (I) AuNPs in dispersion states. (II) Aggregated AuNPs in the presence of NaCl. (III) Aptamers bind to AuNPs and result in re-dispersion of AuNPs. (IV) Aptamers recognize and connect with Candidalysin and release AuNPs. The red spheres represent dispersed gold nanoparticles, while the blue spheres represent aggregated gold nanoparticles. (**B**) Structure of Candidalysin.

Here, we developed a dual-mode biosensor based on AuNPs for screening high-affinity aptamers for Candidalysin, including label-free colorimetric–SERS. As shown in Figure 1, citrate-stabilized AuNPs are well dispersed against aggregation due to the negative capping agent’s electrostatic repulsion, and the UV–vis spectra of AuNPs exhibit a characteristic absorbance peak at 520 nm, while the Raman spectra show a high intensity of Raman characteristic peaks at 1193 cm^−1^ and 1583 cm^−1^. Once NaCl is added, charge-imbalance-induced self-aggregation of AuNPs leads to a significant decrease in absorbance and visible color change, the absorbance peak of the UV–vis spectrum shifts to 680 nm, and Raman characteristic peaks become less distinct. When AuNPs are mixed with an appropriate concentration of aptamer, the aptamer adsorbs on the surface of the AuNPs and protects the AuNPs from NaCl-induced aggregation; the characteristic peaks in the UV–vis spectra and Raman spectra remained unchanged. If the high-affinity aptamer were present, Candidalysin would specifically bind to it to form a complex, and the aptamer would be released from the surface of the AuNPs, causing the aggregation of AuNPs; the UV–vis and Raman spectra reflect the aggregation state of the AuNPs. The concentration of the affinity aptamer is converted into changes in the position and intensity of the absorbance peak, as well as changes in the Raman signals of the AuNPs coupled with Candidalysin. Through the detection of the dual-mode biosensor, we finally found a specific aptamer with high-affinity for Candidalysin from 80 candidates. This work provides a practical workflow to obtain and validate Candidalysin-binding aptamers; our platform is designed not only for detection but also as a screening and validation workflow to discover a new, first-in-class Candidalysin-binding aptamer, and may facilitate future diagnostic and anti-virulence development.

## 2. Materials and Methods

### 2.1. Materials and Reagents

The oligonucleotide library, primers, and Candidalysin toxin (sequence: SIIGIIMG-ILGNIPQVIQIIMSIVKAFKGNK) were procured from Sangon Biotech Corporation, Shanghai, China. The NaCl powder was obtained from Shanghai Aladdin Biochemical Technology Co., Ltd., Shanghai, China. The AuNPs solution was purchased from XFNANO Materials Tech Co., Ltd., Nanjing, China (all from the same batch). The 96-well microplates used were polystyrene, flat-bottomed, and tissue-culture-treated (Corning Incorporated, Corning, NY, USA, cat. no. 3595). All chemicals used in our experiments were of analytical standard and were used without further purification.

### 2.2. Equipment

The UV–vis absorption spectra of the prepared materials were measured using a TECAN microplate reader (model: 30086376; Tecan Trading AG, Männedorf, Switzerland) with a wavelength range from 400 nm to 800 nm. The morphology and structure of the AuNPs were observed using a high-resolution transmission electron microscope (JEM-2100F, JEOL Ltd., Tokyo, Japan). SERS spectra were acquired using a Renishaw InVia Raman spectrometer (Renishaw plc, Wotton-under-Edge, Gloucestershire, UK).

### 2.3. In Vitro Selection of ssDNA Aptamers Against Candidalysin

The SELEX procedure was conducted following previously reported methods [27,28]. The experiment employed a combined approach of positive and negative selection. For the first round, 1 nM ssDNA library and 5 nM biotinylated complementary DNA (cDNA) were added into 250 μL of selection buffer (10 mM Tris-HCl, 0.5 mM MgCl_2_, 20 mM NaCl, pH 7.4) for immobilization. Then, the bulk solution was heated at 95 °C for 10 min and maintained at 25 °C for 30 min after natural cooling to allow for the formation of secondary structure. Following this, the mixture and 250 μL of streptavidin-coated agarose beads were added to a gravity-flow column, and 250 μL of selection buffer was passed through the column ten times, facilitating the immobilization between ssDNA and agarose beads and removing unbound oligonucleotides. Prior to positive selection, 750 μL of bovine serum albumin (BSA) solution was passed through the column to remove sequences with non-specific affinity for Candidalysin, followed by a wash with 250 μL of selection buffer. Finally, 750 μL of Candidalysin was passed through the column to elute the target-bound ssDNAs. The eluted solution was concentrated, desalted, and collected for PCR amplification. The PCR conditions were set as follows: 95 °C for 2 min; 95 °C for 15 s, 58 °C for 30 s, and 72 °C for 45 s, repeated for 10 cycles; and 72 °C for 5 min. The PCR products were then added and passed through a new gravity-flow column in which 200 μL of streptavidin-coated agarose beads had been added. Subsequently, 400 μL of 0.2 M NaCl was added to the column for 10 min to dissociate the DNAs and beads. The pH was neutralized to 7.0–7.5 with a 0.2 M HCl solution, followed by purification through ethanol precipitation. The collected ssDNA served as the secondary library and participated in the next SELEX round. The cycle was repeated 6 times until the affinity of the ssDNA pool for Candidalysin ceased to increase.

### 2.4. Aptamer Synthesis

All aptamers used in this study (Table 1, Supplementary Table S1) were synthesized by Sangon Biotech Corporation, Shanghai, China.

### 2.5. Preparation and Characterization of AuNPs

A high-resolution transmission electron microscope (JEM-2100F, JEOL Ltd., Tokyo, Japan) was used to illustrate the morphology and characterization of AuNPs. Simultaneously, the particle size of AuNPs was determined using the Image J software (version 1.53t) particle analyzer tool. All experiments were performed using AuNPs synthesized in the same batch to ensure valid internal comparisons.

### 2.6. Experimental Procedure

The general procedure for Candidalysin concentration detection is as follows: First, 10 μL of 0.7 M NaCl solution was added to 80 μL of AuNPs solution, incubating for 10 min in a 4 °C refrigerator in the dark; the color of AuNPs changed from red to blue immediately. Then, 10 μL of 10 mM Candidalysin aptamer solution was added and incubated with AuNPs for 100 min, stored at 4 °C in the dark; the color turned to pink gradually. Next, 10 μL of 15.1 μM Candidalysin was added to the aptamer–AuNPs complex for light-protected incubation at 4 °C for 30 min. The entire working scheme is depicted in Figure 1A, and Figure 1B demonstrates the chemical structure of Candidalysin.

### 2.7. Colorimetric Determination of Reaction Solution

The UV–vis spectrum of the reaction solution was evaluated in the range of 400 to 800 nm using a TECAN microplate reader with solvent matrix serving as the control in the 96-well microplates. For NaCl-induced AuNPs aggregation assays, the solvent matrix used for background correction consisted of citrate, PBS, and Tris-HCl. For aptamer incubation experiments, the matrix comprised NaCl, citrate, PBS, and Tris-HCl. For Candidalysin-induced AuNPs aggregation, the matrix included NaCl, aptamer, citrate, PBS, and Tris-HCl. The recorded data was used to generate a working curve with the wavelength as the *x* coordinate and absorbance as the *y* coordinate. The value of the absorbance peak value at 520 nm was documented for the quantification of Candidalysin.

### 2.8. SERS Analysis of Reaction Solution

The SERS spectra were measured using a Renishaw InVia Raman spectrometer, and spectra were collected from 800 cm^−1^ to 1900 cm^−1^. Solutions of dispersed AuNPs, AuNPs–NaCl, and AuNPs–NaCl–Aptamer–Candidalysin were, respectively, tested under a Raman laser after colorimetric determination in a 96-well microplate. Data was verified by subtracting the base line of a blank 96-well microplate.

## 3. Results and Discussion

### 3.1. Characterization of AuNPs

**Figure 2:**
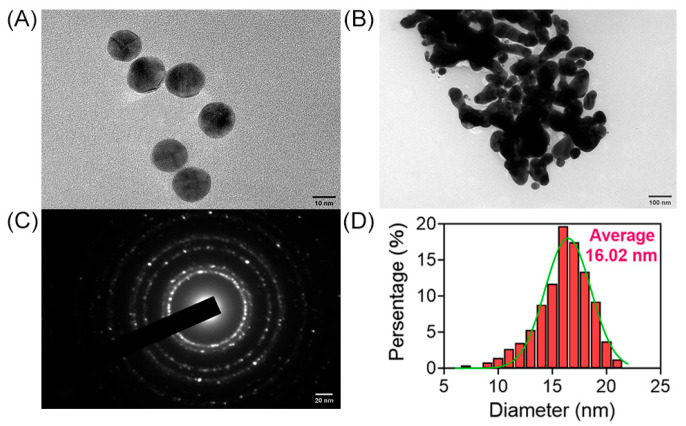
Characterization of AuNPs. HRTEM images of AuNPs in dispersion (**A**) and in aggregation (**B**). (**C**) Diffraction images of AuNPs. (**D**) Particle size analysis of AuNPs.

The morphology and structure of AuNPs were examined using high-resolution transmission electron microscopy (HRTEM), providing images of AuNPs in both dispersed and aggregated states. Figure 2A depicts AuNPs in a dispersed state, with a majority of particles displaying a spherical shape. However, under specific conditions, such as the presence of NaCl, the dispersed AuNPs tend to aggregate into larger particles, as shown in Figure 2B, captured by HRTEM. The diffraction pattern of AuNPs is presented in Figure 2C, while the statistical diameter of AuNPs, approximately 16.02 nm, is demonstrated in Figure 2D.

### 3.2. Mechanism of the Aptamer–AuNPs-Based Colorimetric Biosensor

The mechanism of the Aptamer–AuNPs-based colorimetric biosensor is predicated on the aggregation and dispersion of AuNPs under certain conditions. In a colloidal solution, negatively charged AuNPs exist in a dispersed state. Upon addition of NaCl, citrate-stabilized AuNPs aggregated (blue). Aptamers can adsorb onto AuNPs and stabilize their dispersion, maintaining the red color [18,29]. When Candidalysin was introduced, it bound the aptamer and displaced it from the AuNPs surface, re-enabling aggregation (blue); the shift in UV–vis spectra and the SERS response were then used for quantification, forming different |A_520_′ − A_520_|/A_520_ ratios at varying Candidalysin concentrations (A_520_ and A_520_′ demonstrate absorbance at 520 nm before and after addition of Candidalysin, respectively) [30,31]. This mechanism enables the quantitative determination of Candidalysin.

### 3.3. Optimization of Determination Conditions

**Figure 3:**
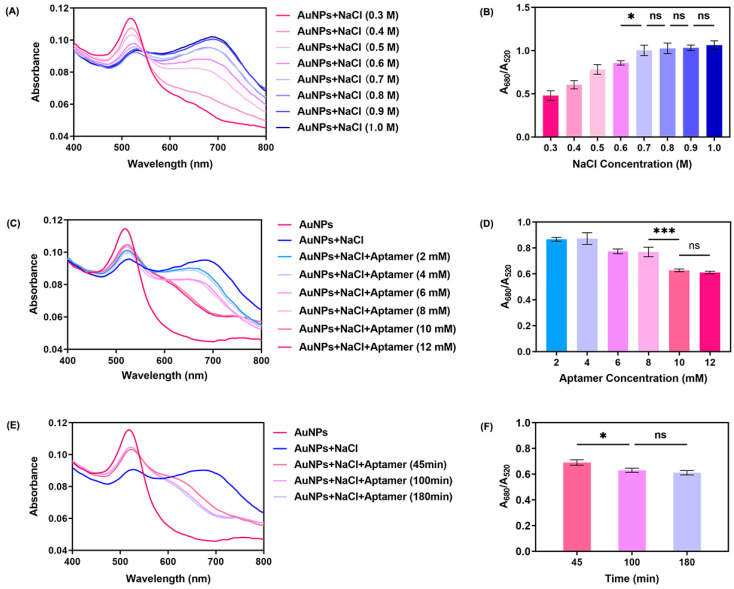
Optimization of NaCl concentration, aptamer concentration, and aptamer incubation time. (**A**) UV–vis spectra of AuNPs under different conditions of NaCl concentration (80 μL of 23.9 μg/mL AuNPs and 10 μL of 0.3 M–1.0 M NaCl). (**B**) A_680_/A_520_ of AuNPs under different conditions of NaCl concentration (n = 3). (**C**) UV–vis spectra of AuNPs under different conditions of aptamer concentration (80 μL of 23.9 μg/mL AuNPs, 10 μL of 0.7 M NaCl, and 10 μL of 2 mM–12mM aptamer). (**D**) A_680_/A_520_ of AuNPs under different conditions of aptamer concentration (n = 3). (**E**) UV–vis spectra of AuNPs after addition of NaCl with time accumulating (80 μL of 23.9 μg/mL AuNPs, 10 μL of 0.7 M NaCl, and 10 μL of 10 mM aptamer). (**F**) A_680_/A_520_ of AuNPs after addition of NaCl with time accumulating (n = 3). Results are expressed as mean ± SD. *p* values were determined by one-way ANOVA and Tukey’s post hoc test, and * *p* < 0.05, *** *p* < 0.001, “ns” denotes no significant difference.

The concentration of NaCl is pivotal in inducing the aggregation of AuNPs within this reaction system. To ascertain the threshold concentration of NaCl necessary to trigger AuNPs aggregation, we scrutinized the UV–vis spectrum of AuNPs (23.9 μg/mL) in the presence of NaCl solutions ranging from 0.3 M to 1 M. As illustrated in Figure 3A, an increase in NaCl concentration leads to a decline in the absorption peak at 520 nm, with the emergence of a new absorption peak at approximately 680 nm. Notably, the spectrum becomes more stable as the concentration escalates from 0.6 M to 1 M, indicating the threshold concentration lies within this range; upon reaching a concentration of 0.7 M, the absorbance spectra stabilize. The state change of AuNPs was quantified using the A_680_/A_520_ ratio. As shown in Figure 3B, no further aggregation of AuNPs occurred after the NaCl concentration reached 0.7 M, leading us to select 0.7 M as the standard NaCl concentration for inducing AuNPs aggregation within our determination system. Hence, our reaction system utilizes 23.9 μg/mL AuNPs and 0.7 M NaCl to achieve optimal AuNPs aggregation.

In our reaction solution, the aptamer plays a crucial role in preserving the charge balance of AuNPs and maintaining their dispersed state. We conducted several investigations to optimize the concentration of the aptamer and its incubation time to ensure that the gold nanoparticles remained in a dispersed state under the protection of the aptamer, unaffected by the charge effects induced by NaCl, which are necessary for the aptamer and AuNPs to achieve maximum immobilization efficiency. A volume of 10 μL of aptamer solution, with concentrations ranging from 2 mM to 12 mM, was added to a system containing AuNPs and 0.7 M NaCl. The absorbance of the system was measured, and the results are shown in Figure 3C. When the aptamer concentration increased to 10 mM, the AuNPs system reached a stable dispersed state. Simultaneously, as shown in Figure 3E, as incubation time increases, the specific absorption peak shifts from 680 nm back to 520 nm; upon reaching an incubation time of 100 min, the reaction nears a saturation, with both the UV–vis spectra and color ceasing to change. Meanwhile, Figure 3D,F illustrate the corresponding changes in the A_680_/A_520_ ratio during aptamer optimization. These results similarly indicate that 10 mM is the optimal concentration for the aptamer, and 100 min is its optimal incubation time. These changes signify the return of the AuNPs to a dispersed state due to the interaction between the aptamer and AuNPs. Consequently, we have selected 10 mM and 100 min as the optimal concentration and immobilization time for the aptamer and AuNPs.

With the introduction of the target Candidalysin sample, the aptamer rapidly recognizes and binds to it, triggering the release and aggregation of AuNPs in the presence of NaCl. As a result, the solution color transitions from red to dark blue, accompanied by changes in the absorption spectrum corresponding to the aggregation of AuNPs. If the aptamer specifically recognizes Candidalysin and forms a complex with it, the protection provided by the aptamer to the AuNPs is lost, causing the AuNPs to return to a dispersed state. Subsequently, the color of the entire reaction system turns dark blue, and the absorbance peak decreases at 680 nm while increasing at 520 nm inversely.

### 3.4. Screening of 80 Aptamers and Construction of the Aptamer–AuNPs Biosensor

**Figure 4:**
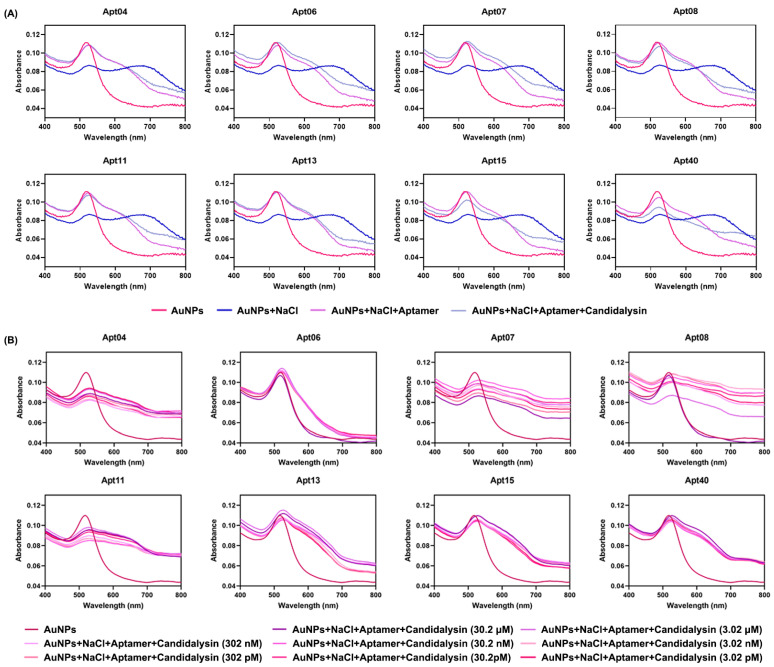
UV–vis spectra of 8 aptamers. (**A**) UV–vis spectra of 8 aptamers we screened out before and after addition of Candidalysin (80 μL of 23.9 μg/mL AuNPs, 10 μL of 0.7 M NaCl, 10 μL of 10 mM aptamer, and 10 μL of 15.1 μM Candidalysin). (**B**) UV–vis spectra of AuNPs with 8 aptamers after adding tenfold dilutions Candidalysin (80 μL of 23.9 μg/mL AuNPs, 10 μL of 0.7 M NaCl, 10 μL of 10 mM aptamer, and 10 μL of Candidalysin solution at concentrations ranging from 3.02 pM to 30.2 μM).

**Figure 5:**
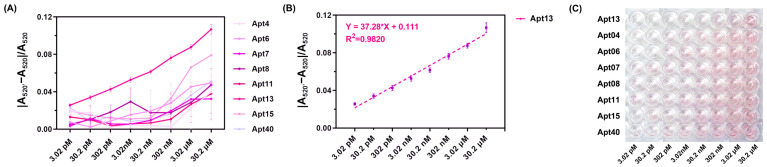
Data processing. (**A**) The ratio of |A_520_′ − A_520_|/A_520_ of eight aptamers. (**B**) Linear regression of Apt13. (**C**) Color changes of reaction systems containing different aptamers upon addition of various concentrations of Candidalysin.

Following the SELEX process, we screened 80 ssDNAs as potential aptamers for final biosensor construction (sequences of the 80 ssDNAs are listed in Supplementary Table S1). As previously mentioned, Candidalysin specifically recognizes and binds to its complementary aptamer, leading to a color change and a shift in the absorbance peak. Utilizing this property, each of the 80 different aptamers was individually incubated with AuNPs for 100 min for preliminary screening. The resulting UV–vis spectra of these 80 aptamers after the addition of 10 μL of 15.1 μM Candidalysin are presented in Supplementary Figure S1. Among these, eight candidates drew our attention due to slight recoveries in solution color and UV–vis spectra. Specifically, upon the addition of Candidalysin, the solution color became more grayish blue, accompanied by a slight increase in absorbance at the 680 nm peak. The sequences of these eight aptamers are detailed in Table 1, and the corresponding absorbance curves are depicted in Figure 4A. Furthermore, these eight aptamers were further tested against different concentrations of Candidalysin, ranging from 3.02 pM to 30.2 μM, to generate the corresponding absorbance spectra and |A_520_′ − A_520_|/A_520_ values, as depicted in Figure 4B and Figure 5A, respectively. Figure 5C depicts the gradual fading of the red color with increasing Candidalysin concentration. Throughout the entire experimental system, the aptamer-to-Candidalysin ratio consistently remained in a state of aptamer excess. Even at the highest target concentration (2.75 μM in the final system), the number of aptamer molecules was 331-fold greater than that of Candidalysin. This condition ensures a linear relationship between the signal response and the target concentration, serving as a crucial prerequisite for achieving a wide dynamic range and high sensitivity.

**Figure 6:**
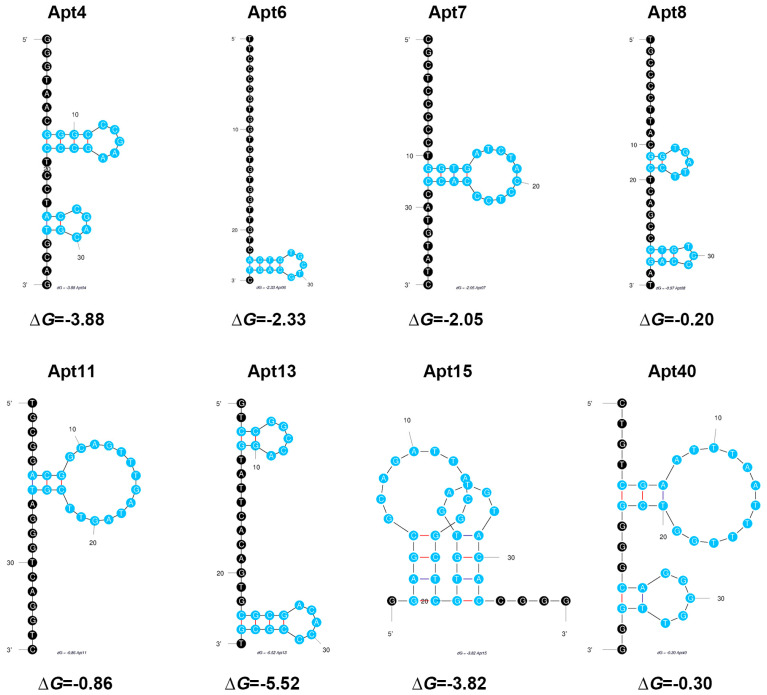
Structure of eight aptamers. The blue color represents the hairpin structure.

According to the results displayed in Figure 4 and Figure 5A, Apt13 emerges as the optimal aptamer due to its high affinity for Candidalysin and its effective binding capacity. The UV–vis spectrum of Apt13 before and after the addition of Candidalysin is shown in Figure 4A. As depicted in Figure 4B and Figure 5A, increasing concentrations of Candidalysin were introduced into the Apt13–AuNPs complex, resulting in distinct UV–vis spectra and varying values of |A_520_′ − A_520_|/A_520_. To further validate the superiority of Apt13 over other aptamers, we explored the ∆*G* and secondary structure of each candidate. Figure 6 illustrates their respective structures. A smaller value indicates a more stable aptamer structure, suggesting that Apt13 is the most likely candidate to form a stable secondary structure with the addition of target Candidalysin. Conversely, Apt8, Apt11, and Apt40 are the least likely to stably combine with Candidalysin due to their small |∆*G*| values. Moreover, as a circular structure, the hairpin loop provides DNA aptamers with higher stability compared to stranded DNA, thus improving the affinity between the aptamer and the target. Consequently, Apt4, Apt13, and Apt15, which have two hairpin loops, may show better specificity than Apt7, Apt8, and Apt11. Meanwhile, because the CG base pair is considered to be more stable than the AT base pair, it is inferred that Apt13, which has six CG base pairs, is more stable than Apt4, which has five base pairs. Leveraging the evidence mentioned above, we utilized Apt13 to develop our colorimetric biosensor. Our analysis revealed a linear relationship (R^2^ = 0.9820) between |A_520_′ − A_520_|/A_520_ and the concentration of Candidalysin using Apt13, as demonstrated in Figure 5B. The lowest quantification limit achieved was 3.02 pM.

### 3.5. SERS Assay to Verify the Above Results

**Figure 7:**
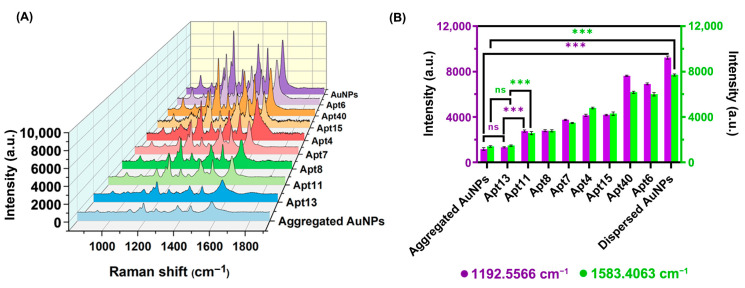
Raman scattering results of 8 aptamers. (**A**) SERS spectra of AuNPs solution (dispersion states), AuNPs–NaCl solution (aggregation states), and 8 AuNPs–NaCl–aptamer–Candidalysin solutions. (**B**) Raman intensity (n = 3) at 1193 cm^−1^ and 1583 cm^−1^ of aggregated AuNPs, dispersed AuNPs, and the eight AuNPs–NaCl–aptamer–Candidalysin solutions. Results are expressed as mean ± SD. *p* values were determined by one-way ANOVA and Tukey’s post hoc test. *** *p* < 0.001, “ns” denotes no significant difference..

As mentioned above, we conducted a preliminary screening of eight aptamers by analyzing the UV–vis spectra of AuNPs before and after the addition of Candidalysin (Supplementary Figures S1 and S2) and refined the concentration of Candidalysin from 3.02 pM to 30.2 μM. Based on the linear relationship between |A_520_′ − A_520_|/A_520_ and Candidalysin concentration, we tentatively identified Apt13 as the optimal aptamer for Candidalysin. To further validate our findings, SERS was employed to test and compare the affinity preference among the eight aptamers by comparing SERS spectra of AuNPs solution (dispersion states), AuNPs–NaCl solution (aggregation states), and eight AuNPs–NaCl–aptamer–Candidalysin solutions. As shown in Figure 7A, all Raman spectra from 800 cm^−1^ to 1900 cm^−1^ of AuNPs in different environments have Raman peaks at 1193 cm^−1^ and 1583 cm^−1^, indicating these two wavelengths are the characteristic Raman peaks of AuNPs; the difference is the dispersed AuNPs solution exhibited higher intensity at 1193 cm^−1^ and 1583 cm^−1^ compared to the aggregated AuNPs solution. Leveraging this, the reaction system with eight different aptamers was irradiated under a laser to analyze and compare the Raman spectra. From Figure 7A, it is evident that Apt13 displayed the closest intensity at 1193 cm^−1^ and 1583 cm^−1^ to the aggregated AuNPs, and the Raman spectrum of the AuNPs–NaCl–Apt13–Candidalysin solution is the most similar to that of the aggregated AuNPs solution. This supports the conclusion that Apt13 recognizes Candidalysin and induces the aggregation of AuNPs, thus establishing itself as the optimal candidate among the eight aptamers. Quantitative analysis in Figure 7B further demonstrates the Raman intensity at 1193 cm^−1^ and 1583 cm^−1^ of aggregated AuNPs, dispersed AuNPs, and the eight AuNPs–NaCl–aptamer–Candidalysin solutions, indicating that Apt13 may bind Candidalysin and induce the aggregation of AuNPs. Consequently, SERS validated the outcomes of the colorimetric method and provided evidence supporting the affinity between Candidalysin and Apt13.

### 3.6. Specific Validation of Aptamers

**Figure 8:**
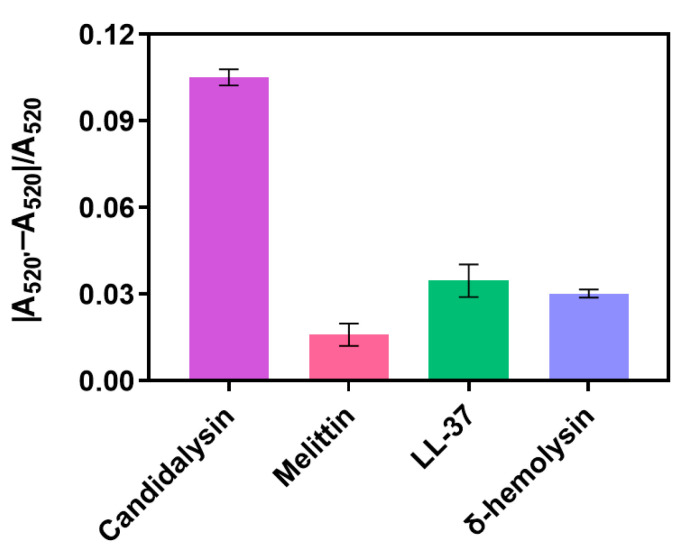
|A_520_′ − A_520_|/A_520_ ratio of Candidalysin and three other toxins with the concentration of 30.2 μM.

To confirm the specificity of Apt13, Candidalysin and Melittin, LL-37, and δ-hemolysin were employed as controls. They were separately tested at a concentration of 30.2 μM with Apt13. The absorbance was recorded, and the results of |A_520_′ − A_520_|/A_520_ are, respectively, displayed in Figure 8. The results indicate that Melittin, LL-37, and δ-hemolysin do not induce a significant alteration in |A_520_′ − A_520_|/A_520_. In contrast, Candidalysin was specifically captured by Apt13, demonstrating the excellent affinity between Apt13 and Candidalysin.

### 3.7. Advantages and Limitations of the Biosensor

Owing to the unique optical characteristics of AuNPs, the sensitivity of the constructed biosensor surpasses traditional quantitative methods for Candidalysin detection, with the lowest detection concentration of 3.02 pM. We applied a colorimetric–SERS dual-mode method to verify the optimal aptamer for Candidalysin with the highest affinity, thereby optimizing the specificity of our biosensor. Furthermore, compared to ELISA, HPLC, or other traditional Candidalysin-detecting approaches [32,33], our biosensor does not require laborious pre-experimental treatment and large precision instruments, making the quantification of Candidalysin more convenient. Compared to traditional single-mode screening methods, our dual-mode (colorimetric–SERS) platform offers crucial advantages in both principle and application: the two physically distinct detection mechanisms enable cross-validation during aptamer screening to minimize false positives, while in practical use the colorimetric mode provides instrument-free visual detection for rapid on-site screening, and the SERS mode delivers high-specificity analysis in complex samples via molecular fingerprinting, together creating a reliable “screening–confirmation” system that balances speed with accuracy. However, our work does have limitations. Real samples are complex and often contain interfering chemicals like saline solutions, which can affect the states of AuNPs, resulting in increased experimental error and inaccuracy of results. Further efforts are needed to optimize the surface modification of AuNPs and pre-preparation of samples, and strict quality control will also improve the accuracy and reliability of the biosensor.

## 4. Conclusions

In this work, we have developed a novel method for determining Candidalysin concentration using an aptamer–AuNPs-based colorimetric biosensor. Initially, we screened 80 different aptamers to identify the one with the best fit and highest affinity for Candidalysin. We employed two distinct approaches and performed secondary structure analysis to achieve this goal. The colorimetric method served as the primary screening tool, and through this method, we found that Apt13 was capable of inducing slight aggregation and corresponding color change of AuNPs. Importantly, a linear relationship between Candidalysin concentration and |A_520_′ − A_520_|/A_520_ was established within the range of 3.02 pM to 30.2 μM, leading us to select Apt13 for the construction of our colorimetric biosensor. Concurrently, surface-enhanced Raman spectroscopy (SERS) was also conducted as a supplementary method.

## Figures and Tables

**Table 1:** Sequences of eight aptamers we screened out.

Name	Sequence (5′ to 3′)	Length (NT)
Apt4	GGGTAACGGGCCCGAAGCCCTCCTACCGACGTGCAG	36
Apt6	TTCCCCGTGGTCTGTGGTTGTCACTGTGCTGCAGTC	36
Apt7	CGCTCCCCCTGGTGATCTACCTCCCACCCATGTATC	36
Apt8	TGCCCCTTACGGTGATTCCTCAGCCCTGTCCCAGAT	36
Apt11	TGCGGACGGCAGTTTGATAGTTCGTAGGGTCAGGTC	36
Apt13	GTCCGGCCAGGTATTCACAGTGCGCGACACCCGCGT	36
Apt15	GGAGCGCAGATTACGGCTCGTGTGATGTACACCGGG	36
Apt40	CTGTCGAATTTAATTTTGGTCGGGGCAGGGGTTGGG	36

## Data Availability

The original contributions presented in this study are included in the article/Supplementary Materials. Further inquiries can be directed to the corresponding authors.

## Supplementary Materials

The following supporting information can be downloaded at: https://www.mdpi.com/article/10.3390/bios16010035/s1, Figure S1: UV–vis spectra of Apt01–Apt40; Figure S2: UV-vis spectra of Apt41–Apt80; Figure S3: Optimization of concentration of NaCl, concentration of aptamer and incubation time; Figure S4: Dose–response curve of 8 aptamers; Figure S5: KD of 8 aptamers; Table S1: Sequences of the 80 ssDNAs.

## Abbreviations

The following abbreviations are used in this manuscript:

AuNPs	Gold nanoparticles
HRTEM	High-resolution transmission electron microscopy
UV-vis	Ultraviolet–visible spectroscopy
SERS	Surface-enhanced Raman scattering
LSPR	Localized surface plasmon resonance
