# Supplementary material for: Discovery of the High-Affinity Aptamer for Candidalysin Using a Dual-Mode Colorimetric–SERS Platform
Source: Biosensors (Basel). 2026 Jan 2;16(1):35. doi: 10.3390/bios16010035 (PMC12838935; doi:10.3390/bios16010035)
Supplement: Supplementary file 1 [file biosensors-16-00035-s001.zip › biosensors-3995312-supplementary.pdf]

biosensors

MDPI

Supplementary Materials

Table S1. Sequences of the 80 ssDNAs.

Name	Sequence (5′ to 3′)	Length (NT)
Apt1	GGGGGAGGGTGGGTGGGAGTCGATGCGCTTGTGATG	36
Apt2	AAGCGATACAAGTCTCCCCCATAAATCACCAGCTTA	36
Apt3	TTTCGTGTCCACCCATTTGCACCAGCGCTTTGTTTT	36
Apt4	GGGTAACGGGCCCGAAGCCCTCCTACCGACGTGCAG	36
Apt5	CCAGCAGTACGGACAACGATTCAAAGTGGCCCTCCT	36
Apt6	TTCCCCGTGGTCTGTGGTTGTCACTGTGCTGCAGTC	36
Apt7	CGCTCCCCCTGGTGATCTACCTCCCACCCATGTATC	36
Apt8	TGCCCCTTACGGTGATTCCTCAGCCCTGTCCCAGAT	36
Apt9	TTCGTCACCCTTTGCTTGTTGCCTGTCCCAACGTAA	36
Apt10	CCACTCACTCGATTTCGCTGCGCATGTTCCCCGTTT	36
Apt11	TGCGGACGGCAGTTTGATAGTTCGTAGGGTCAGGTC	36
Apt12	TCGGCACCCCGTGTTGGACGGCTCGTCCCCCTGACA	36
Apt13	GTCCGGCCAGGTATTCACAGTGCGCGACACCCGCGT	36
Apt14	CCATCATCGTCCCGCTTACCCGTGCTCTGTTTCTCA	36
Apt15	GGAGCGCAGATTACGGCTCGTGTGATGTACACCGGG	36
Apt16	GCCTGTTAGATTGGCGCGGGGGGAGGGGTTGGGTTGTGTTATGCGTGCTAC-CGTGA	56
Apt17	GTGTTATGCGTGCTACCGTGAGATCGGAAGAGCACACGTCTGAACTCCAG-TCACCG	56
Apt18	AGGGGGGACATCTGGATTGTTGGCCGTGCGGATACGGTGTTATGCGTGC-TACCGTG	56
Apt19	ATGTACCAAGGACTGCTTAGGATTGCGATGTAGTATGTGTTATGCGTGC-TACCGTG	56
Apt20	TGTCTCTCGGTTGGTCTTGGGGGAAGGGGTTGGGTTGTGTTATGCGTGCTAC-CGTG	56
Apt21	CGCACATGGCTTTAGAGGTTCCATAGGGTACACTCTGTGTTATGCGTGC-TACCGTG	56
Apt22	ACGGGTAGGCTTATGCTTGTCGTAAACGCTCCGGATGTGTTATGCGTGC-TACCGTG	56
Apt23	ACCACGGTCGGTCCCTCGGTATTCTGGTCCCTGCTCTGTGTTATGCGTGC-TACCGT	56

Apt24	GTGTTATGCGTGCTACCGTGGTGTTATGCGTGCTAC-CGTGGTGTTATGCGTGCTAC	56
Apt25	TTTTCGGGGGACTGCTCGGGATTGCGGATACGATGTGTTATGCGTGCTAC-CGTGAG	56
Apt26	GCCTGTTAGATTGGCGCGGGGGGAGGGGTTGGGTT	35
Apt27	AGGGGGGACATCTGGATTGTTGGCCGTGCGGATACG	36
Apt28	ATGTACCAAGGACTGCTTAGGATTGCGATGTAGTAT	36
Apt29	TGTCTCTCGGTTGGTCTTGGGGGAAGGGGTTGGGTT	36
Apt30	CGCACATGGCTTTAGAGGTTCCATAGGGTACACTCT	36
Apt31	ACGGGTAGGCTTATGCTTGTCGTAAACGCTCCGGAT	36
Apt32	ACCACGGTCGGTCCCTCGGTATTCTGGTCCCTGCTCT	37
Apt33	TTTTCGGGGGACTGCTCGGGATTGCGGATACGAT	34
Apt34	GGGGTAAGGGGTTGGGTTCTTGCTAAGGCTTCGTTG	36
Apt35	CTCGCGTTGGGTTTGGCTATGGGGGAGGGGTTGGGGTA	38
Apt36	GCAGGCAAGGCTCTACTGACCCGTTGCTTGATCAAC	36
Apt37	GTGGTATTCAAGTGGGCTTCAGGGGGAGGGGTTGGG	36
Apt38	GTGTTATGCGTGCTACCGTG	20
Apt39	ACTCCCACCTTTATTGAGGGCGGTGACGGGTTCCCTC	37
Apt40	CTGTCGAATTTAATTTTGGTCGGGGCAGGGGTTGGG	36
Apt41	CTCAGGCTTGAAGGGGCTGCATGTGGTGGTGCGACT	36
Apt42	TGAGTCCGGTGTTGGCACTGATCGCCGAATTTCCGT	36
Apt43	GGCGTCCACACGCTATGTGCTCAGGGCATCTCTCTT	36
Apt44	CTCGTCGCTCCGTGGCCTAAGAGGGTTCGCCCCTGT	36
Apt45	CTCCACGCCTAAGCATCCCAGAGGGGCCCTCAGGGA	36
Apt46	TGGGGCACGGCATTACCCGCATGTTCAGCTCGTAGC	36
Apt47	AGCCGTTCCACTCGAGATAACAGTGTACCTCTGTCC	36
Apt48	CCACAACATCCTCGTCCCCGCCTGCTTACTGCGGCG	36
Apt49	GTCATGGTGATTTTAGCGTCGCGATTCTTGGATATC	36
Apt50	CCACTTCACGCGGCACTCGCAACCTGGCCCGCTTGA	36
Apt51	GCACCCGCTAACTCGTAGTCCCCAGCCACTCGACTA	36
Apt52	CCGTTCACCCCGTTCCCTTCAGCATGTGGCTGCACC	36
Apt53	TAGGCGCGGAGACTAGGGAGCAACCTAATCCTCCAA	36
Apt54	AGGCTCGACCTGCTGTCCCGCGGTCTTCATGACCCT	36
Apt55	GGCCGCAGTCTCATCTTGGCTGTGACTACGCGGTTC	36
Apt56	TCCTCGGGTTCCCGTGGTGAGCTCCACCATGATCCT	36

Apt57	ACAACCGTGGATCGAGAAACCTACACTTTTCCGCCC	36
Apt58	CGTCGGAAGCCAAGGCACCTAGACTCCGGGTGGTTC	36
Apt59	CCAGCCAATTGGCGTGCGCTTGCGAGCCGCACTCTG	36
Apt60	CAGTCCGTCCTCATCAACCGCATGAAGAACCGGTGT	36
Apt61	CGATCGAGCAACCAGTAATGCATCCGCCACCATGCGTGCTACCGTGA-GATCGGAAG	56
Apt62	TGCACGGGACGGGTTTATAAGCTCCAGTGGTAGTCGGTGTTATGCGTGC-TACCGTG	56
Apt63	ACGCGCCGGCCGTCCCTCCTTGCCTGGC-TACAATTCGAAGGGATCCGTGTTATGCG	56
Apt64	GCAGCCGGGCTTGCCGGTCATCTTGGACGCTGTTACTTGTTATGCGTGCTAC-CGTG	56
Apt65	GACCTGATGGGGAGACTTACCGGGTGGCACTCCAGTGTTATGCGTGCTAC-CGTGAG	56
Apt66	TCCCGCTCATCTACAAAGTGAGATCTTCAGCTCGCTGTGTTATGCGTGCTAC-CGTG	56
Apt67	TGGGCGTGTACCCATCTAAAGATCCTCTATCACTGCGTGTTATGCGTGCTAC-CGTG	56
Apt68	GCTGCTCACCATCAACGGGAAGCAATCCGGCGCGGAGTGTTATGCGTGC-TACCGTG	56
Apt69	CGCCCAGGCAGTCTCGTTGGTAGTCGAATCCGCGGCTGTGTTATGCGTGC-TACCGT	56
Apt70	TCCTCCAGCTGGTCTCAATCATACCAACGGAGGGTAGTGTTATGCGTGC-TACCGTG	56
Apt71	CGTGCTGACTAGAGAGCTCCAGCAGGTGGCGGGAAACCTGCTCAC-GGGCCCTTGAATT	58
Apt72	CTCAATACCGGTCTCTCATAGCGACGTGCGAACGTC	36
Apt73	CTTGGGGCGTCCTGGTCACGACCTGGCTAGGGCCAT	36
Apt74	TACGCGGAGTTGTCAAAAACCGAAGCTCGGAAATGT	36
Apt75	TCTCGCTCGCGCGGAGCCCCCGCCCCCGGCACACCG	36
Apt76	GGGCAAACGGTGCGTCTGCATTCAGTGTGCATCAT	35
Apt77	CAATTGCGCTACCTTGGTGGCAAAGTGGAACTGGAC	36
Apt78	GCTACTTGTGTGATCCCCTTACTGCTGGCACGGCGT	36
Apt79	CACCTGGGTCAGCGACCGGAGCCCTTGTGAGAATGA	36
Apt80	GCCTTGGCCAGCCCGGGGAACACGCCGCCCCCGCATT	37

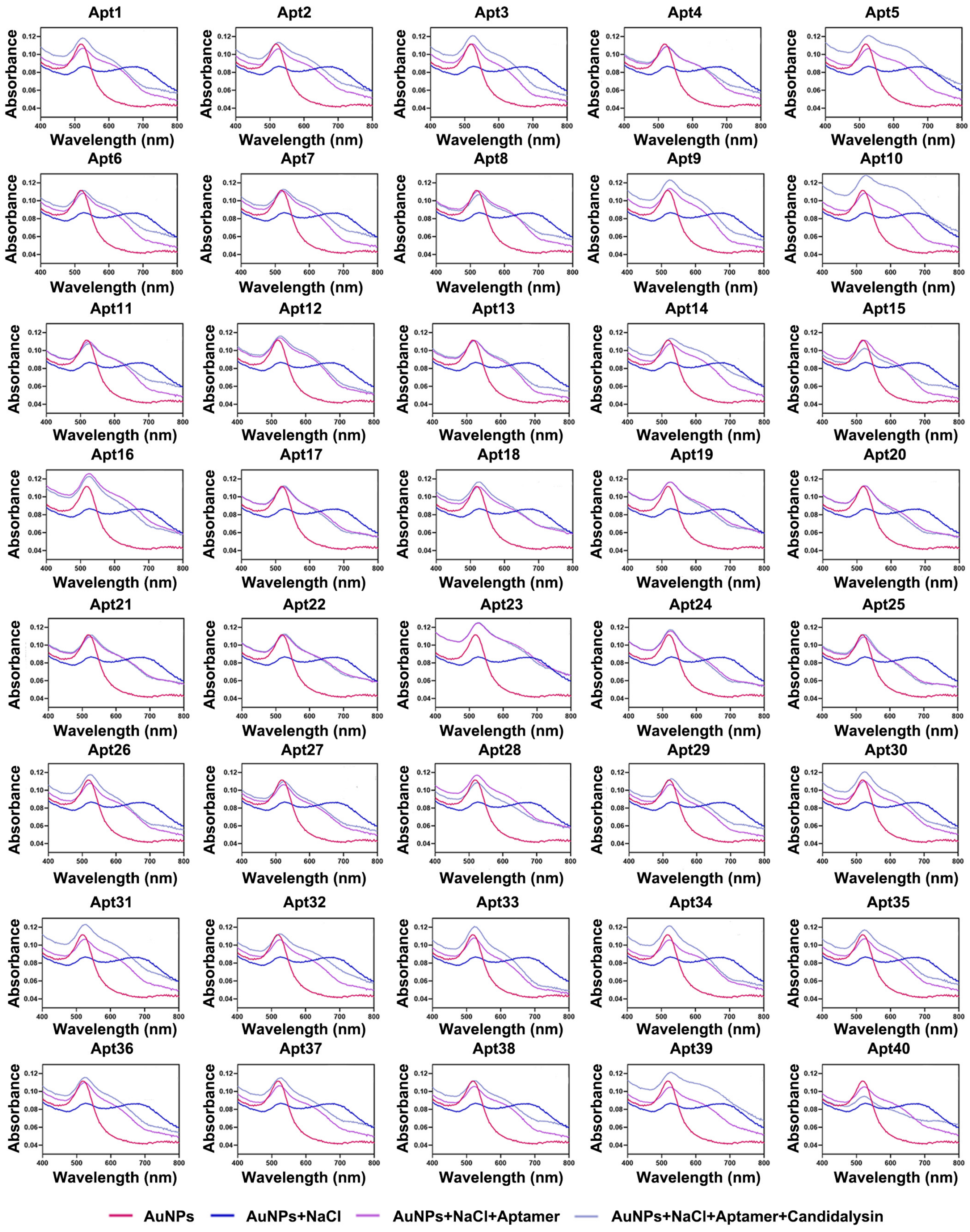

Figure S1. UV-vis spectra ofApt01-Apt40 before and after addition of Candidalysin (80 μL of 23.9 μg/mL AuNPs, 10 μL of 0.7 M NaCl, 10 μL of 10 mM aptamer and 10 μL of 15.1 μM Candidalysin).

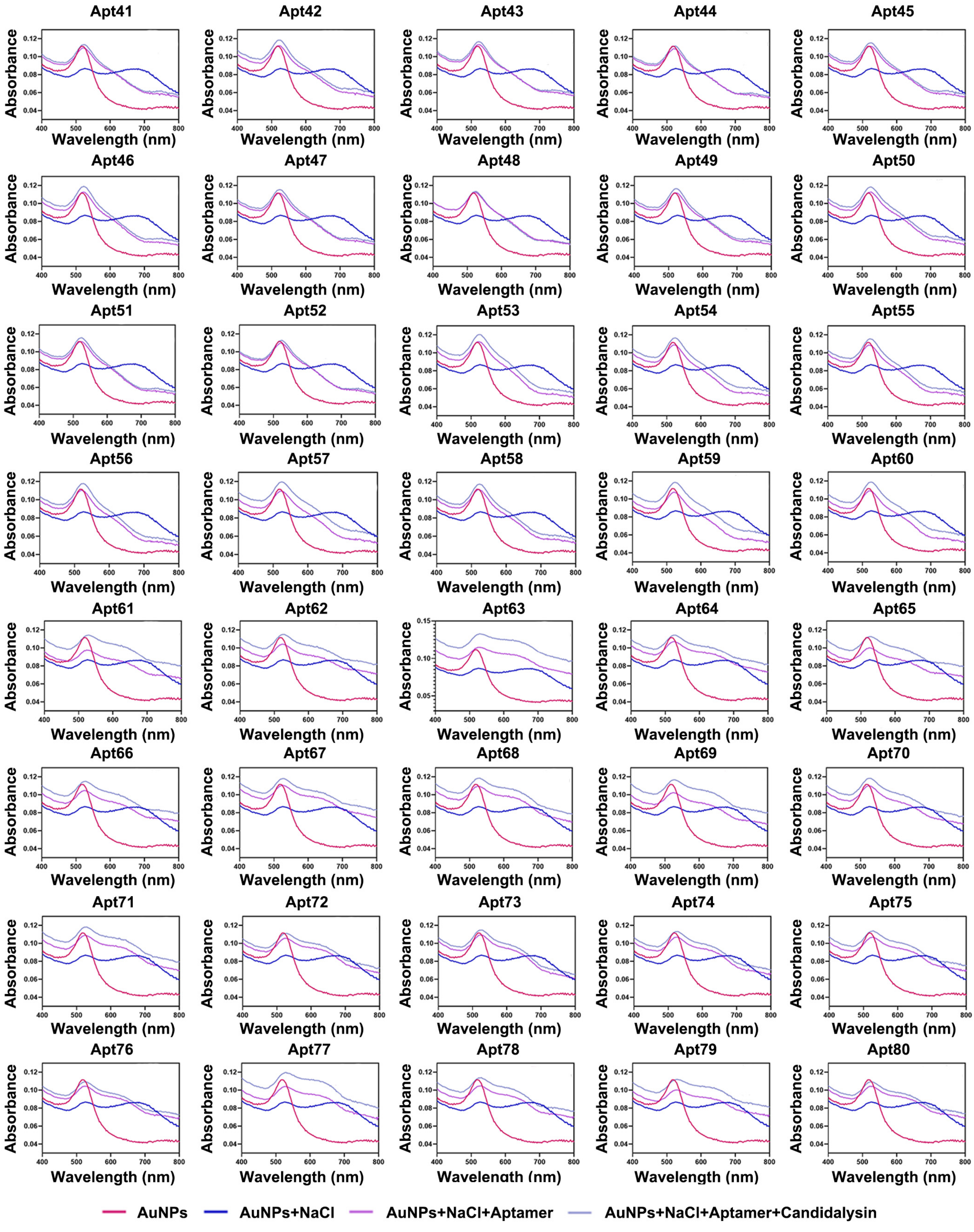

Figure S2. UV-vis spectra ofApt41-Apt80 before and after addition of Candidalysin (80 μL of 23.9 μg/mL AuNPs, 10 μL of 0.7 M NaCl, 10 μL of 10 mM aptamer and 10 μL of 15.1 μM Candidalysin).

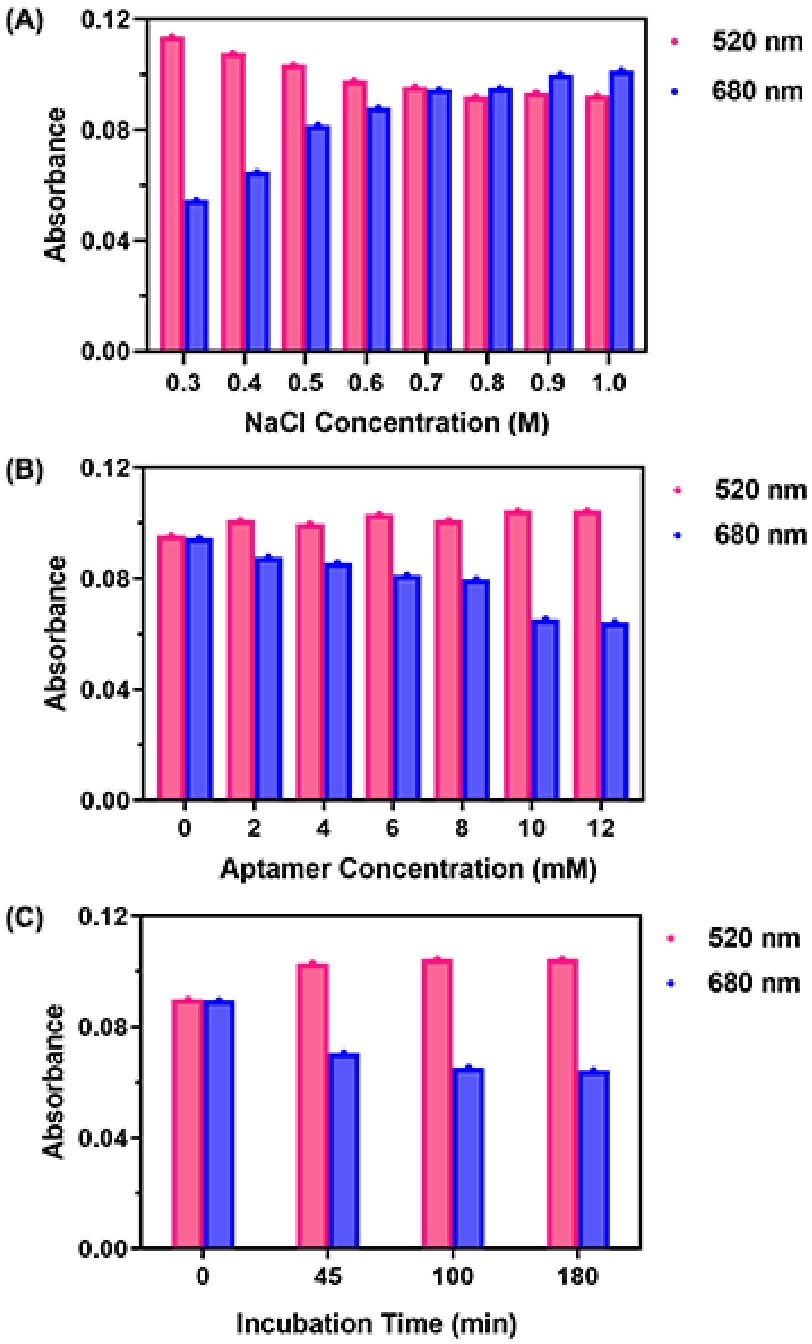

Figure S3. Optimization of concentration of NaCl, concentration of aptamer and incubation time. (**A**) Dose–response curve of AuNPs under different conditions of NaCl concentration (80 μL of 23.9 μg/mL AuNPs and 10 μL of 0.3 M–1.0 M NaCl). (**B**) Dose–response curve of AuNPs under different conditions of aptamer concentration (80 μL of 23.9 μg/mL AuNPs, 10 μL of 0.7 M NaCl and 10 μL of 2 mM – 12mM aptamer) (**C**) Dose–response curve of AuNPs after addition of NaCl with time accumulating (80 μL of 23.9 μg/mL AuNPs, 10 μL of 0.7 M NaCl and 10 μL of 10 mM aptamer).

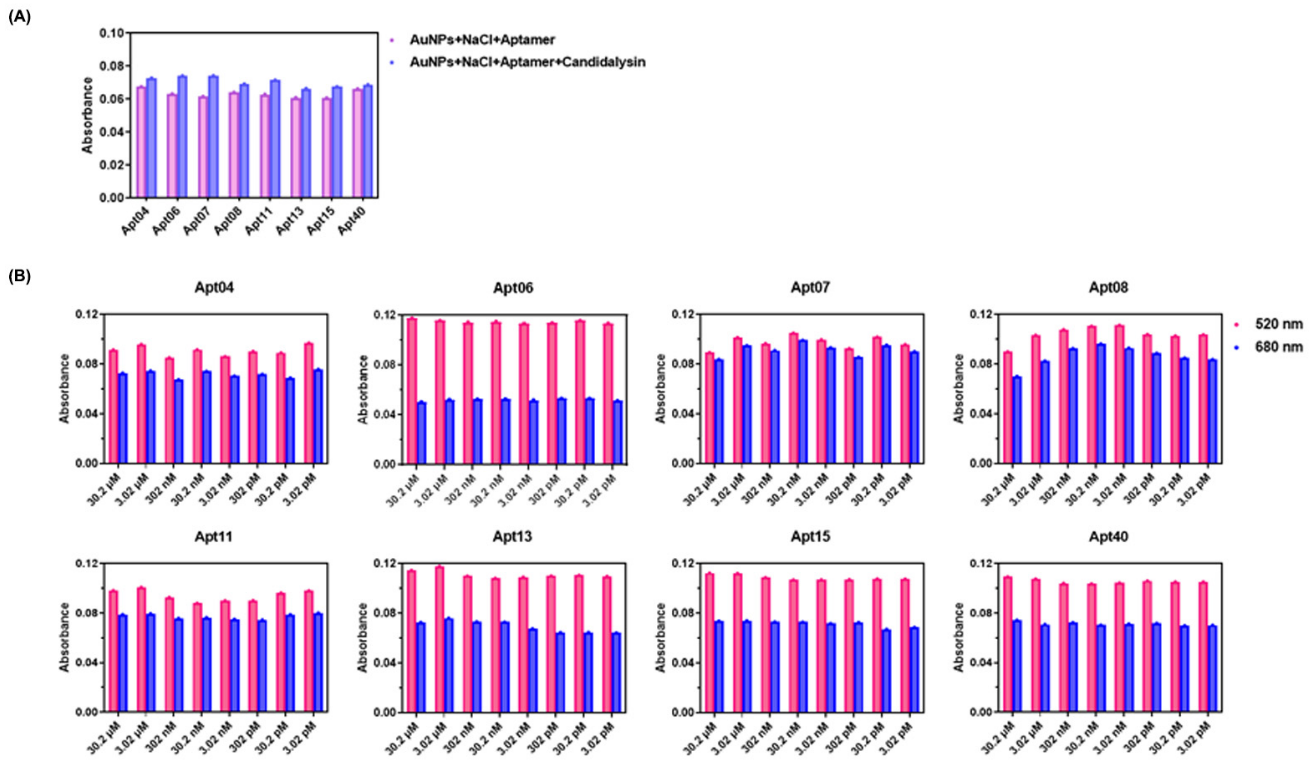

Figure S4. Dose–response curve of 8 aptamers. (**A**) A_{680} of 8 Aptamers we screened out before and after addition of Candidalysin (80 μL of 23.9 μg/mL AuNPs, 10 μL of 0.7 M NaCl, 10 μL of 10 mM aptamer and 10 μL of 15.1 μM Candidalysin). (**B**) A_{520} and A_{680} of AuNPs with 8 aptamers after adding tenfold dilutions Candidalysin (80 μL of 23.9 μg/mL AuNPs, 10 μL of 0.7 M NaCl, 10 μL of 10 mM Aptamer and 10 μL of Candidalysin solution at concentrations ranging from 3.02 pM to 30.2 μM).

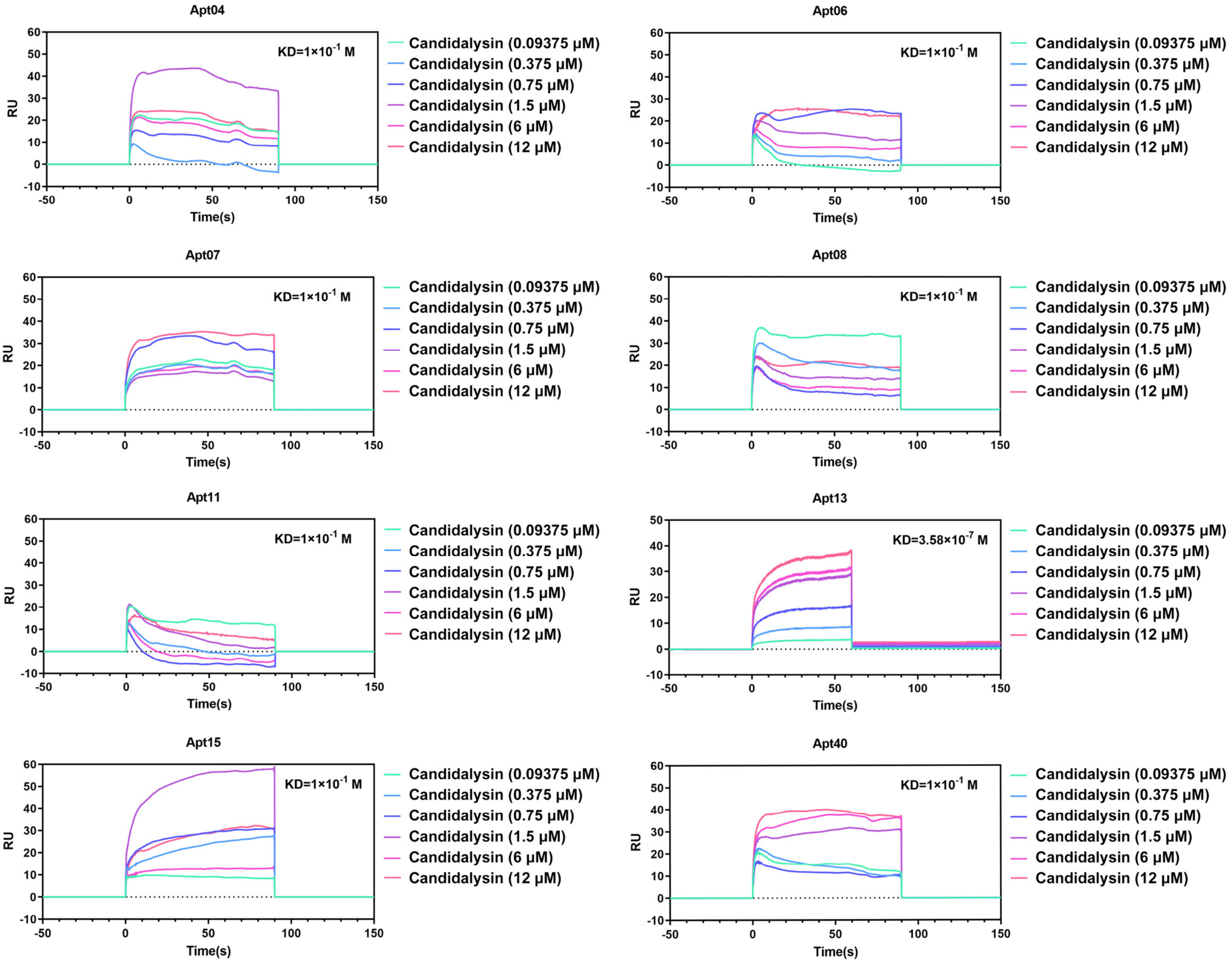

Figure S5. KD of 8 aptamers.
